# Corticostriatal Plasticity Established by Initial Learning Persists after Behavioral Reversal

**DOI:** 10.1523/ENEURO.0209-20.2021

**Published:** 2021-03-10

**Authors:** Sanchari Ghosh, Anthony M. Zador

**Affiliations:** 1 Cold Spring Harbor Laboratory, Cold Spring Harbor, NY 11724; 2 Cold Spring Harbor Laboratory School of Biological Sciences, Cold Spring Harbor Laboratory, NY 11724

**Keywords:** animal behavior, auditory-motor association, coticostriatal plasticity, reversal learning, stimulus-action association, synaptic plasticity

## Abstract

The neural mechanisms that allow animals to adapt their previously learned associations in response to changes in the environment remain poorly understood. To probe the synaptic mechanisms that mediate such adaptive behavior, we trained mice on an auditory-motor reversal task, and tracked changes in the strength of corticostriatal synapses associated with the formation of learned associations. Using a ChR2-based electrophysiological assay in acute striatal slices, we measured the strength of these synapses after animals learned to pair auditory stimuli with specific actions. Here, we report that the pattern of synaptic strength initially established by learning remains unchanged even when the task contingencies are reversed. Our findings reveal that synaptic changes associated with the initial acquisition of this task are not erased or overwritten, and that behavioral reversal of learned associations may recruit a separate neural circuit. These results suggest a more complex role of the striatum in regulating flexible behaviors where activity of striatal neurons may vary given the behavioral contexts of specific stimulus-action associations.

## Significance Statement

We have established that learning a specific auditory-motor association establishes a distinct pattern of plasticity in the tonotopic projection from auditory cortex to auditory striatum in mice. The sign of this association can be read out postmortem, with nearly perfect fidelity, using electrophysiological measurements from a single acute brain slice. We then trained another cohort of mice to reverse this association after the initial training period, and measured the plasticity pattern in this circuit. Surprisingly, even after learning the new association successfully, the corticostriatal plasticity pattern represented the initial association, acquired over two weeks ago. Our results have implications for the role of corticostriatal plasticity in forming stimulus-action associations and understanding the neural basis of learning in adaptive behaviors.

## Introduction

One of the key neural mechanisms for adaptive behavior involves changes in the strengths of specific synaptic connections. Different behaviors involve different circuits, and thus recruit changes at different synaptic connections. In fear conditioning paradigms, for example, the association of a tone and a foot-shock induces freezing behavior that is mediated by long-term potentiation or LTP (Malinow and Malenka, 2002) at specific synapses that convey auditory information to the amygdala (LeDoux, 2000; Rumpel et al., 2005). Similarly, in barn owls the alignment of visual and auditory spatial maps for sound localization is mediated by specific synaptic connections in the inferior colliculus (Feldman and Knudsen, 1997). Although synaptic plasticity is thought to mediate many forms of learning, the specific loci of the synaptic changes have been experimentally established in only a handful of behavioral paradigms.

A hallmark of animal adaptation is that it is an ongoing and continual process, typically occurring not just once, but often throughout the lifetime of the animal. For example, a tone that predicts a shock one day might not predict it the next. It might seem intuitive that unlearning of such a previously formed association, “extinction,” would involve simply overwriting or erasing the synaptic changes underlying the initial tone-shock association. Indeed, optogenetic potentiation and depotentiation of auditory inputs to the amygdala can mediate bidirectional activation and deactivation of cue-induced freezing behavior (Nabavi et al., 2014). At the behavioral level, however, extinction of sound-induced freezing behavior does not appear to involve simple erasure of the initial memory, but rather inhibition of the freezing response by other brain structures (Quirk et al., 2000). Similarly, chronic prism placement alters the topography of synaptic connections in the inferior colliculus of the barn owl (Knudsen and Brainard, 1995; Feldman and Knudsen, 1997), but the connections formed early in life persist even after they are no longer functionally expressed (Linkenhoker et al., 2005). By contrast, stimulation-induced persistent LTP in the hippocampus can be reversed if animals are exposed to novel environments as opposed to their familiar arena, suggesting that the same ensemble of synapses may be re-used in new environments (Xu et al., 1998). Thus, the extent to which ongoing behavioral adaptation to a changing environment recruits the same synapses as in the initial learning remains an open and complex question, the resolution of which may depend on the specific circuits and behaviors involved.

Many brain regions involved in learning are parts of the basal ganglia, which consist of distinct nuclei. The chief input nucleus, the striatum, integrates inputs from various cortical and sub-cortical areas. The motor striatum is broadly implicated in movement control (Kreitzer and Malenka, 2008; Klaus et al., 2019), and stimulating a particular subset of striatal neurons, the “direct pathway” neurons, in the anterior dorsal striatum (Kravitz et al., 2010) promotes contralateral movement. By contrast, the same stimulation in the auditory striatum (Guo et al., 2018), or in the dorsomedial striatum (Tai et al., 2012), only introduces a choice bias in the context of the behavioral task. Unlike the motor striatum, in which neuronal activity is finely tuned to movement initiation (Cui et al., 2013), neurons in the auditory striatal neurons mainly encode stimulus features during sound presentation (Guo et al., 2018). Moreover, recent studies show that unlike anterior striatum, dopaminergic projections to the posterior striatum originate from substantia nigra pars lateralis (SNL) and may not signal the commonly believed reward prediction error (Menegas et al., 2015, 2018).

Here, we have used an auditory two alternative choice (2-AC) decision task to study learning associated synaptic changes in a part of the posterior striatum, the auditory striatum. Training rats to perform an auditory discrimination task (Znamenskiy and Zador, 2013) induces potentiation of corticostriatal synapses, forming a spatial plasticity gradient along the tonotopic gradient of auditory inputs to the auditory striatum (Xiong et al., 2015). The sign of this gradient, which can be read out in acute slices of the auditory striatum, is determined by the precise stimulus-response association learned: in animals trained to associate low-frequency stimuli with a left decision, the sign of the gradient is opposite to that in animals trained to associate low-frequency stimuli with a right decision (Xiong et al., 2015). These and other observations suggest that strengthening connections between sensory cortices and their striatal targets facilitates appropriate action selection after learning (Kreitzer and Malenka, 2008; Lee et al., 2015; Guo et al., 2018). In this study, we exploit our high-resolution understanding of the synaptic changes elicited by acquisition of this behavior to test whether reversal of the stimulus-response contingencies leads to reversal of the corresponding synaptic strengths. We find that the plasticity gradient established by the initial training in this auditory task is not modulated bidirectionally but instead remains stable even after the contingencies are reversed.

## Materials and Methods

### Animals and viruses

All procedures were conducted in accordance with the institutional animal use and care policies of Cold Spring Harbor Laboratory. C57 Black6J male mice were obtained from Jackson laboratories and housed in a temperature-controlled and moisture-controlled room with 12/12 h light/dark cycle. Viruses used for anatomic tracing experiments, AAV2.1.CAG.GFP and AAV2.1.CAG.tdTomato were ordered from University of Pennsylvania Vector Core. AAV2.9.CAG.Channelrhodopsin virus for optogenetic stimulation was obtained from UNC Vector core.

### Surgical procedures and injections

For performing stereotaxic injections, mice were anaesthetized with a cocktail of ketamine (60 mg/kg) and medetomidine (0.5 mg/kg) and immobilized on a stereotaxic set up. After sterilization with 70% alcohol and numbing with subcutaneous injection of lidocaine (2 mg/kg), the skin and tissue overlying the left auditory cortex was dissected to expose the temporal bone. To cover the entire primary auditory cortex, two injections were made perpendicularly to the brain surface roughly 2 and 2.5 mm caudal to the temporoparietal suture, and roughly 1 mm below the ventral edge. Each injection was made at two depths (400 and 600 μm) releasing approximately 80 nl of virus at each depth.

### Transcranial intrinsic optical imaging

Mice were anesthetized with ketamine (60 mg/kg) and medetomidine (0.5 mg/kg) and immobilized in a stereotaxic setup. After sterilization with 70% alcohol and numbing with subcutaneous injection of lidocaine (2 mg/kg), a portion of the scalp on the top of the cranium was removed and a headbar was attached to the exposed skull using Metabond adhesive (Parkell, S380), further secured using dental cement (Lang, Jet denture repair powder/liquid). After 2–3 d of recovery, the animals were anesthetized using isofluorane (2.5% isofluorane + O_2_ at 0.1 l/min) and immobilized in the stereotaxic set up using the headbar. After sterilization with 70% alcohol and numbing with subcutaneous injection of lidocaine (2 mg/kg), the skin and tissue overlying the left auditory cortex was removed exposing the bone surface. For transcranial imaging, the bone was thinned using a low-speed dental drill. The exposed skull was kept moist during the imaging session using 1.5% agar in PBS. Additional anesthesia was provided by injecting chlorprothixene (0.7 mg/kg) and the mouse was then transferred for intrinsic optical imaging to a custom-built microscope set up. During the imaging, the mouse was kept lightly anaesthetized using isofluorane (1% isofluorane + O_2_ at 0.1 l/min) while placed on a temperature regulated heating pad to maintain the body temperature close to 35°C. Intrinsic signal images were acquired using a CCD camera (Vosskuehler 1300QF) after illumination using red LED (615 nm). In order to evoke stimulus responses in the auditory cortex, 1-s pure tone pips of were played at an interval of 30 s. The frequencies chosen were 4 and 32 kHz, each being presented at least 15 times at 80 dB to map the low-frequency and high-frequency responsive regions of the auditory cortex (Bakin et al., 1996; Bathellier et al., 2012). The acquired images were analyzed to depict normalized difference of reflectance in response to the stimulus [(prestimulus – poststimulus)/prestimulus]. The location of specific tone-responsive regions in these images were registered with respect to the image of the surface vasculature acquired using blue LED (488 nm). These maps were subsequently used to perform tonotopic tracing experiments.

### Behavioral training and apparatus

For training animals on the tonecloud task, mice were deprived of water for 23 h at the end of which they were given 1–1.5 ml of water. After 2 d, the animals were introduced to the behavior boxes for training and were given water only via training. However, animals were never deprived of access to water for >23 h. Water-deprived animals were trained in custom sound-booths by Industrial Acoustics Company containing a custom-built behavioral arena (20 × 20 × 20 cm). This consisted of three ports located on one wall with inter port distance of 5.5 cm (center to center). The height of the port was 2.5 cm from the floor. The side walls of the arena had perforations aligned to the speakers located just outside the walls. Water was delivered through the ports via 19-Gauge stainless steel tubes connected to rubber tubing (SILASTIC) and the flow was controlled via solenoid valves (Lee Company). The valve opening times were calibrated at regular intervals to ensure accurate delivery of 0.5 and 2.5 μl of water from the center and side ports, respectively. LEDs located just above the water ports were used to provide “Go” cues. The auditory stimuli used were high-frequency and low-frequency toneclouds that consisted of trains of short overlapping pure tones drawn from either a high (20–40 kHz) or low (5–10 kHz) octave, up to 500 ms long. These toneclouds were designed by the MATLAB protocol and delivered through the speakers that were calibrated described before (Jaramillo and Zador, 2014). The behavior system was automated and controlled through custom software written in MATLAB to operate the state machine interface of the behavior control module, bpod (https://sanworks.io/shop/viewproduct?productID=1027).

In stage 1 of training, animals poked at the center port in response to a steady center port light-the Go cue for trial initiation. Holding at center port for 50 ms of prestimulus delay successfully initiated a trial and triggered delivery of the sound stimulus and a small reward (0.5 μl) at the center port. A steady light at the correct side port (depending on the frequency content of the sound stimulus) signaled the mouse where to go next for an additional 2.5-μl water reward within the trial duration of 10 s. A new trial started when the animal reported its choice or if the trial time elapsed. There were no punishments for incorrect choices or early withdrawals at this stage. The animals were promoted to stage 2 when they completed >100 successfully rewarded trials within one session in stage 1. In stage 2, both the side port lights were turned on after stimulus delivery to signal the animal that it was time to report a choice and no longer signaled the correct port. At this stage, animals were given a white noise punishment for incorrect trials and early withdrawals. The animals moved on to stage 3 after two sessions of >100 completed trials each. The stage 3 is where animals spent maximum time in training. At this stage, the center port light still provided a Go cue for trial initiation but side port lights stayed off. Early withdrawals were punished with a 1-s timeout and incorrect choices with a 4-s timeout in addition to the white noise. At this stage the prestimulus delay was also increased to 250 ms. Animals were either trained to pair low-frequency toneclouds with a leftward movement and high-frequency toneclouds with rightward (referred to as low-left) or vice versa (referred to as low-right). The training contingency for each animal (low-right or low-left) was randomly predetermined by the experimenter. Animals were trained to the performance criteria of higher than 80% in four to six consecutive sessions before proceeding to recording experiments. For animals performing the reversal task, reversal of contingency was introduced after the same performance criteria as above while maintaining task parameters of stage 3. These animals were then subsequently trained to the same performance criteria in the opposite contingency before proceeding to recording experiments.

### Slice experiments

Mice were first anaesthetized with a cocktail of ketamine (60 mg/kg) and medetomidine (0.5 mg/kg) then perfused with ice-cold artificial CSF (aCSF) bubbled with 95% O_2_ and 5% CO_2_. The mouse was then rapidly decapitated and the brain was removed from the cranium and placed in ice-cold cutting buffer also bubbled with 95% O_2_ and 5% CO_2_. It was then transferred to the stage of a vibratome kept submerged in ice-cold cutting buffer (110 mm choline chloride, 25 mm NaHCO_3_, 25 mm D-glucose, 11.6 mm sodium ascorbate, 7 mm MgCl_2_, 3.1 mm sodium pyruvate, 2.5 mm KCl, 1.25 mm NaH_2_PO_4_, and 0.5 mm CaCl_2_) continuously bubbling with 95% O_2_ and 5% CO_2_. The temperature of the entire set up was maintained at 4°C. The brain was then cut into coronal slices of 250-μm thickness until we reached the canonical slice chosen for our striatal recording. Once the ideal slice was cut, it was quickly transferred into a holding chamber containing continuously aerated aCSF (127 mm NaCl, 25 mm NaHCO_3_, 25 mm D-glucose, 2.5 mm KCl, 4 mm MgCl_2_, 1 mm CaCl_2_, and 1.25 mm NaH_2_PO_4_, aerated with 95% O_2_ and 5% CO_2_) at 32°C. The slice was allowed to recover for ∼30 min and then maintained at room temperature at which the recordings were performed. After recovery, the slice was carefully transferred to the recording set up. CNQX was added to a final concentration of 50 μμ in aCSF and delivered through the perfusion system for inactivating glutamatergic synaptic transmission at corticostriatal synapses.

### Electrophysiology recordings and analysis

Local field potentials (LFPs) were recorded using Axopatch 200B amplifiers (Axons Instruments, Molecular Devices) using thin-walled glass pipettes of resistance 2–3 MΩ filled with filtered aCSF. Light pulses were delivered through a light guide microscope illumination system (Lumen Dynamics) modified to accept a blue laser (473 nm, Lasermate Group) in place of the lamp. The laser beam was focused onto the sample through the 60× objective during recordings, with an illumination field of 350 μm in diameter. Each light pulse was 0.5 ms at 1 Hz, and each recording was an average of approximately ten trials. To minimize the contribution of rundown on the estimation of the plasticity gradient within the striatal slice, recording locations were selected randomly for each slice. For quantification of the channelrhodopsin-2-evoked LFP (ChR2-LFP), each averaged trace was normalized to the peak of the first component. A line was fitted to 10% to 90% of the postsynaptic depolarization phase, whose slope provides the ChR2-LFP for that recording site. For each slice, the ChR2-LFP slopes across sites were rescaled from 0 to 1, with the smallest ChR2-LFP set to zero and the largest to 1. For each animal, this normalized mean ChR2-LFP slope for each position along the tonotopic axis was plotted and the plasticity gradient was defined as the slope of the linear fit to these data for that animal. Precaution was taken to select approximately the same slice from every animal to maintain consistency across experiments and only one slice was used from each animal. To overlay the plasticity gradient maps across animals, the striatal maps for each experiment were aligned to each other using the center of the recording area (as estimated by mean *x* and *y* coordinates of recording sites).

### Data analysis and statistics

All behavior and electrophysiology data were acquired and analyzed using custom designed software written in MATLAB. Wilcoxon rank-sum test was performed to test the significance of difference between learning induced plasticity gradients in animals trained on the tonecloud and reversal-tonecloud tasks. Wilcoxon Signed Rank tests were performed to compare training times and performance analysis before and after reversal of animals on the reversal task. Kruskal–Wallis test was performed to detect presence of significant differences in the ChR2-LFP slopes along the dorsoventral axis of the striatum.

## Results

To assess the synaptic changes associated with acquisition and subsequent reversal of stimulus-response contingencies, we tested the effect of reversal learning on the plasticity gradient of corticostriatal projections in mice. In rats, this gradient is such a sensitive measure of learning contingencies that it can reveal, with 100% accuracy, whether an individual subject has been trained to associate a high-frequency stimulus with a left or a right choice (Xiong et al., 2015). Because the present experiments were conducted in mice, we first confirmed that they can also be rapidly and reliably trained to perform the 2-AC tonecloud task (Chen et al., 2019; see also Guo et al., 2018; Jaramillo and Zador, 2014). We then tested whether they could be reliably trained to reverse the contingencies on which they had initially been trained. Next, we assessed whether projections from mouse cortical area A1 to striatum are organized in a tonotopic fashion. Taking advantage of this tonotopic organization of the corticostriatal projection, we used an electrophysiological assay to assess strength of these synapses along the tonotopic axis. This ability to read out the synaptic correlate of the learned association provides a unique opportunity to probe the changes in this circuit after behavioral reversal. Finally, using the reversal paradigm, we show that the plasticity gradient established at auditory corticostriatal synapses after the initial learning phase persists even after successful reversal learning. Our results suggest that the site of plasticity engaged during reversal may differ from that engaged in the initial learning of the task.

### Acquisition time for initial association and reversal are comparable

**Figure 1.**
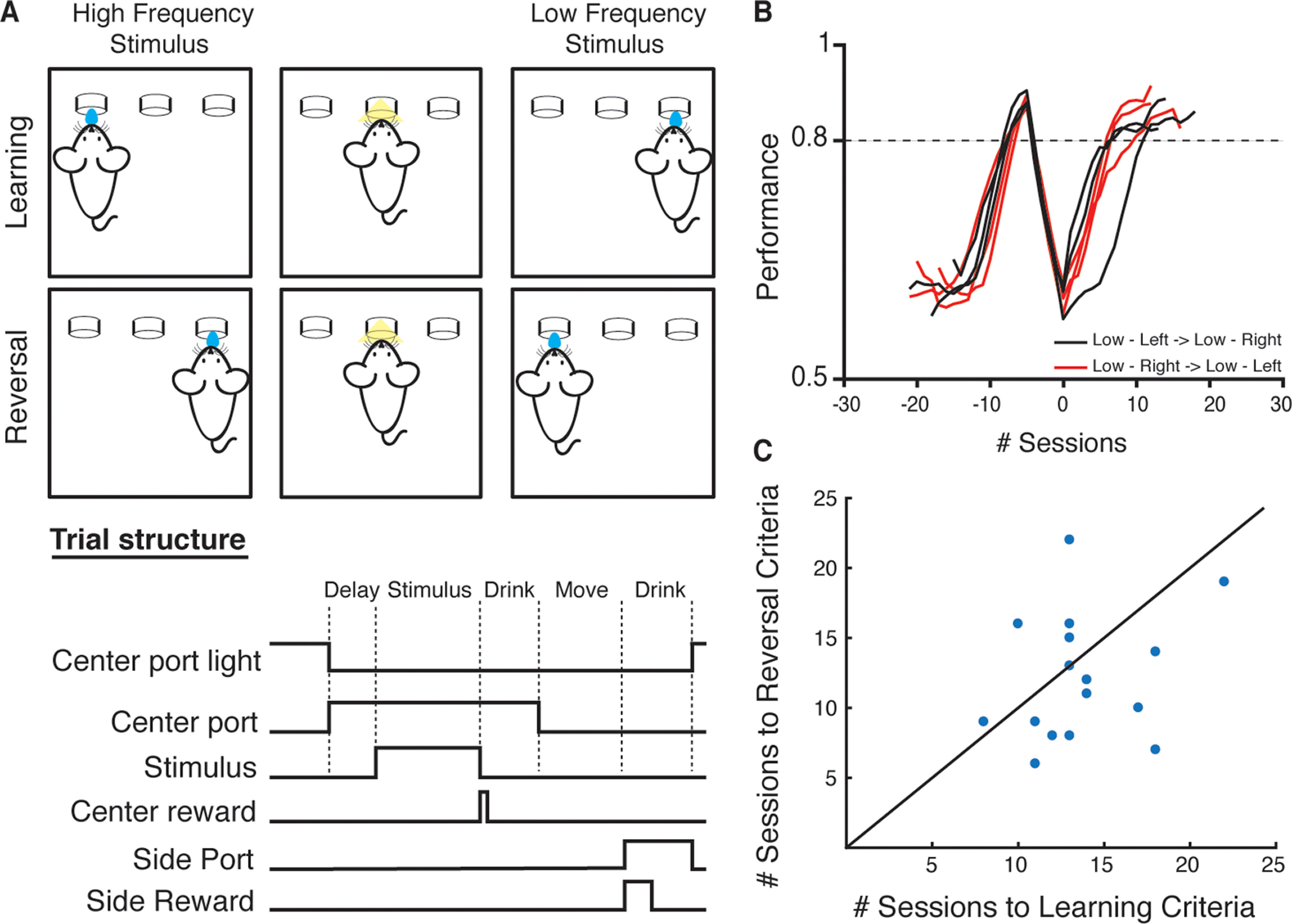
Training mice on a reversal paradigm based on 2-AC frequency discrimination. ***A***, Schematic of the reversal paradigm using the tonecloud task for mice (top). Animals are first trained on one contingency, e.g., to pair a low-frequency tonecloud with reward on the right referred to as “low-right” (“learning”). Once they reach the performance criteria, the training contingency is reversed, requiring the same animal to now pair a low-frequency tonecloud with reward on the left, or “low-left” (“reversal”). The trial structure (bottom) shows the sequence of events in a single typical “correct” trial in the task. ***B***, Example learning curves of mice trained in the reversal paradigm (black, low-left → low-right, *n* = 3 and red, low-right → low-left, *n* = 3), where 0 denotes the start of training on the reversed contingency. The performance of the animals is smoothed over three sessions for this plot. ***C***, Animals require a comparable number of sessions to reach performance criteria during initial learning as during reversal.

The auditory 2-AC task, adapted from a related task developed for rats (Znamenskiy and Zador, 2013), required that subjects discriminate between low and high “tonecloud” stimuli, and report their choice by going to either the left or right choice port (Fig. 1*A*). Subjects initiated a trial after the Go cue (light “on” at center port) was provided. On each trial, the stimulus consisted of a train of short overlapping pure tones drawn from either a low (5–10 kHz) or high (20–40 kHz) octave. Subjects were required to listen to the entire stimulus (500 ms) before reporting their choice; at the end of the stimulus, they were rewarded with a small drop of water (0.5 μl) at the center port, to encourage them to remain in the center port for the duration of the stimulus. After withdrawal from the center port, subjects were required to choose between a left and a right reward port, depending on the frequency content of the stimulus (Fig. 1*A*, top). Mice readily learned this task over a period of two to three weeks.

We next established a reversal paradigm in which subjects trained to criterion on one association (low-left or low-right) were then trained to reverse this association (Fig. 1*A*, bottom). To avoid overtraining subjects on one contingency, and thereby potentially increasing the difficulty in re-training them on the reversed contingency, we established a relatively lax performance criterion of >80% correct per session. After four to six sessions of >80% performance, we reversed the stimulus- response contingency. In the sessions immediately following reversal, all subjects show a marked decrease in performance, often performing well below chance, after which performance increased to levels comparable to the original contingency (Fig. 1*B*). On average, subjects required a similar number of sessions to reach the fixed performance criteria [12.9 ± 0.8 vs 12.5 ± 1.2 (SEM), *p* = 0.61, Wilcoxon signed-rank test; Fig. 1*C*]. Thus, mice could be reliably trained to perform both the basic tonecloud task and the reversal.

### Auditory corticostriatal projections in mice show tonotopy

**Figure 2.**
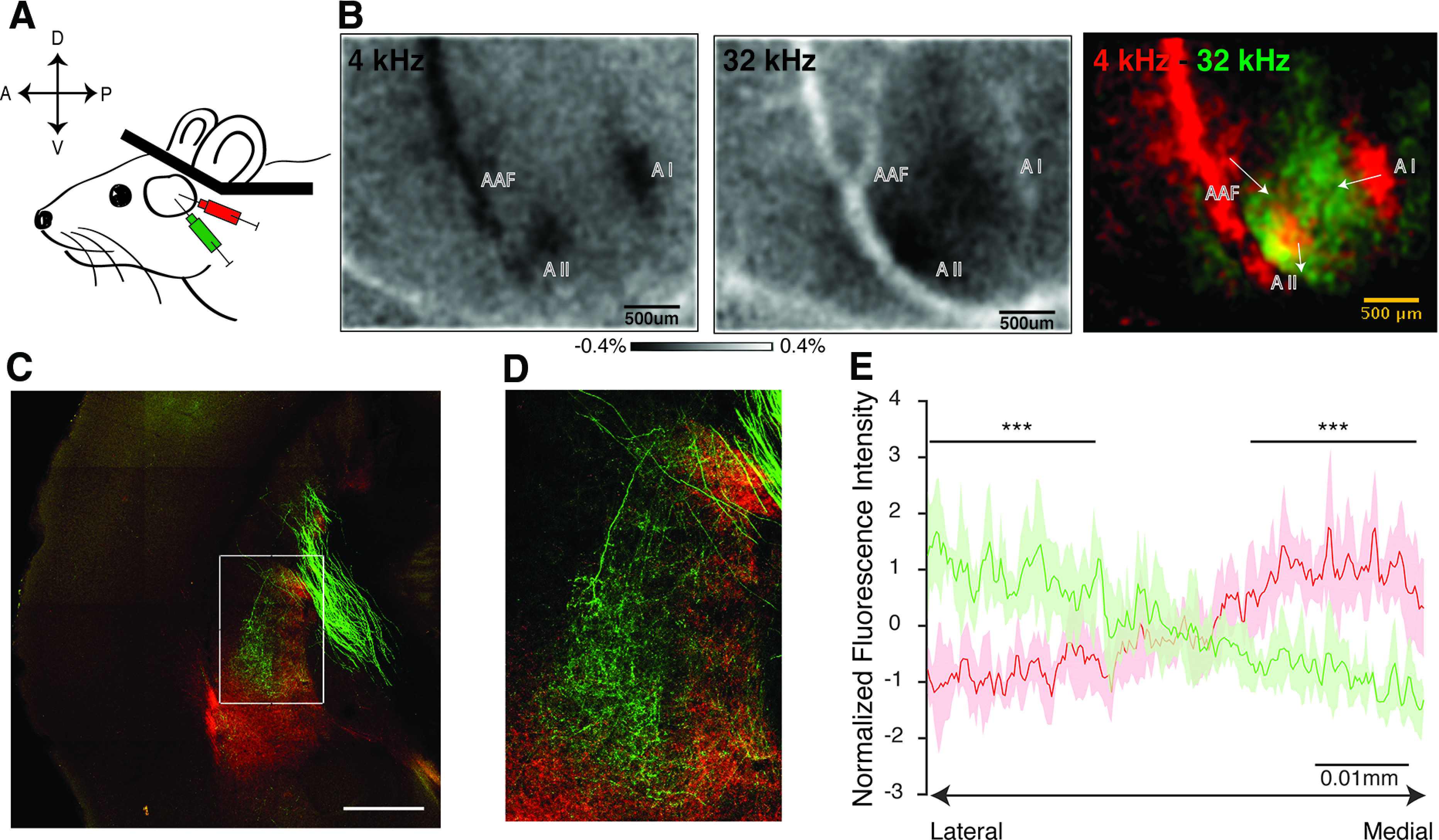
Auditory corticostriatal projections in mice are tonotopically organized. ***A***, Intrinsic optical imaging of the auditory cortex in a head-fixed mouse through a window of thinned bone. ***B***, Intrinsic optical images in response to pure tones of 4 and 32 kHz (left and middle) are shown for the example animal whose histology is shown later. The data shows high and low frequency loci as determined by the mean relative normalized change in reflectance between pre-stimulus period and during stimulus presentation repeated 15×. Composite image showing tonotopy in auditory cortex where green corresponds to high frequency and red to low frequency. The arrows indicate overall tonotopic gradient (low to high) in the individual tone responsive areas. ***C***, Tonotopic separation of A1 projections into auditory striatum in a single example section showing high frequency A1 projects more laterally and low frequency A1 projects more medially (white rectangle). Scale bar = 500 μm. ***D***, Enlarged view of auditory striatum (white rectangle) from C showing tonotopic separation. ***E***, *Z*-score normalized fluorescence intensity of auditory corticostriatal projections plotted along the medio-lateral axis. The data represents mean and std. dev. of fluorescence intensity across 5 slices obtained from 2 animals after dual injections; paired t-test shows significant differences in red and green fluorescence intensity at the medial and lateral ends (*p* = 5.6 × 10^−5^, and *p* = 1.9 × 10^−5^ respectively; *** indicates *p* < 10-4). Additional data is available in Extended Data Figure 2-1.

The mouse auditory system is organized tonotopically (Hackett et al., 2011; Kaas, 2011; Kanold et al., 2014). This feature allows measuring neural activity and plasticity in specific tone- responsive regions (Bathellier et al., 2012; Higgins et al., 2010; Moczulska et al., 2013) . We mapped the tonotopic projections from primary auditory cortex to auditory striatum. We first performed intrinsic optical imaging of the auditory cortex through a thinned bone preparation in a mouse (Bakin et al., 1996; Bathellier et al., 2012), in response to pure tones of 4 and 32 kHz (Fig. 2*A*). The intrinsic signals elicited by these stimuli consistently revealed three regions, which we identified as A1, A2, and AAF (Fig. 2*B*), and subsequently mapped to the brain surface using the vasculature as guidance. We then performed small focal injections in high-frequency and low-frequency regions of A1, using AAV1-CAG-GFP and AAV1-CAG-tdTomato, respectively (Fig. 2*A*; Extended Data Fig. 2-1). Inspection of coronal sections of the auditory striatum revealed a tonotopic organization of the afferent cortical projections (Fig. 2*C*). Fibers from the low-frequency region of A1 terminated more medially, whereas those from the high-frequency region terminated more laterally (Fig. 2*D*,*E*). These experiments reveal a tonotopic projection in the mouse from A1 to the auditory striatum, which can be observed in standard coronal slices.

10.1523/ENEURO.0209-20.2021.f2-1 Extended Data Figure 2-1 Injection site in primary auditory cortex confirming expression of tdTomato (top) and GFP (bottom). These images confirm little to no overlap of viral infections at the cortical injection site. Scale bar: 500 μm. Download Figure 2-1, TIF file.

### Training induces a tonotopic gradient of synaptic strength in the auditory striatum

Striatal plasticity has been previously implicated in skill learning and associative learning (Pasupathy and Miller, 2005; Yin et al., 2005; Cox and Witten, 2019). In rats, acquisition of a frequency-dependent auditory task establishes a gradient of synaptic strength along the tonotopic gradient in striatum (Xiong et al., 2015). We tested whether learning the tonecloud task also resulted in a stereotyped gradient of corticostriatal synaptic strength in the mouse striatum.

**Figure 3.**
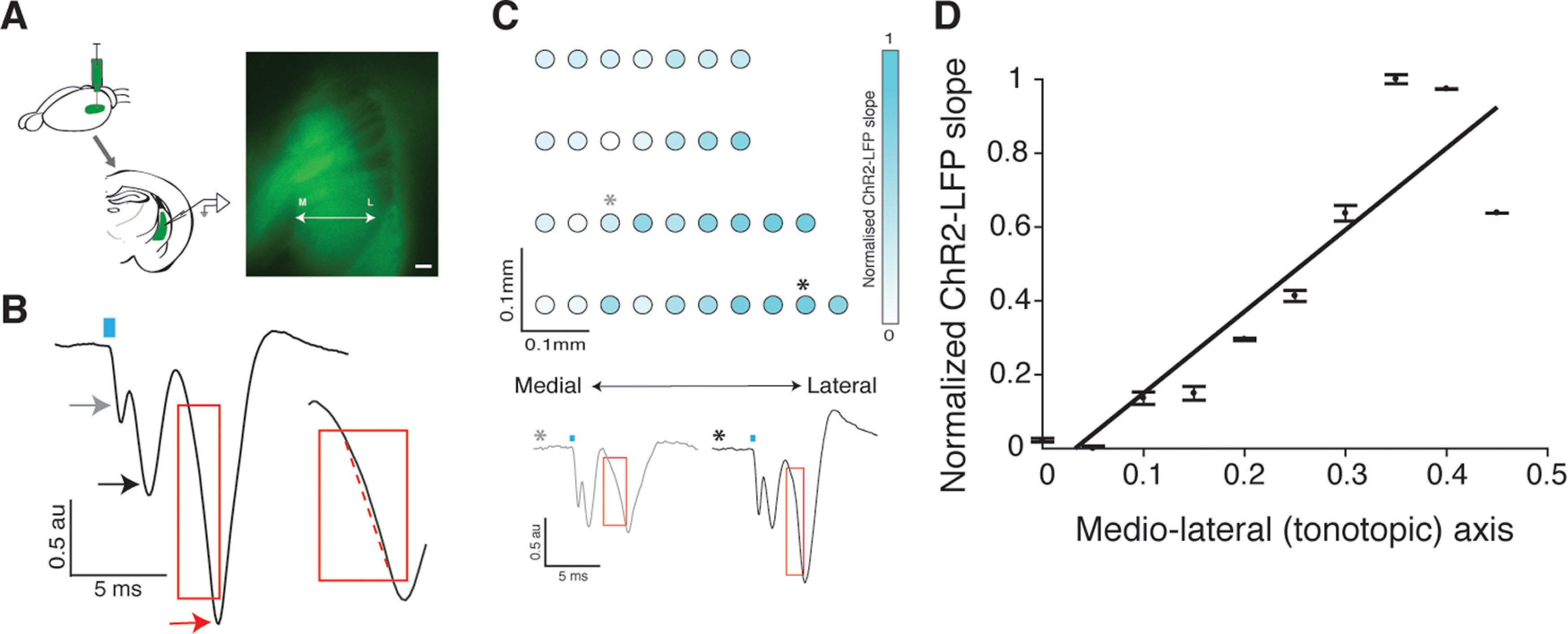
ChR2-LFP slope measurements reflect the learning induced plasticity gradient. ***A***, AAV9-ChR2 is injected in auditory cortex and recordings are obtained from acute coronal slices of auditory striatum exhibiting ChR2-expressing corticostriatal fiber terminals. Right, Example of an acute slice showing ChR2-GFP expressed in corticostriatal fibers. ***B***, Example trace of ChR2-LFP from one position in the slice. Gray arrow indicates a light artifact often observed soon after laser stimulation (blue rectangle). Black arrow indicates the depolarization of ChR2-expressing corticostriatal fibers. Red arrow indicates the postsynaptic response of downstream striatal neurons. The response is normalized to the fiber depolarization, and the normalized ChR2-LFP slope is calculated from the postsynaptic component (red rectangle). Inset, Calculation of ChR2-LFP slope by fitting a line (dotted red line) to the postsynaptic component. ***C***, Representative image showing distribution of individual normalized ChR2-LFP slopes along the tonotopic axis of the left auditory striatum of an example animal trained on low-left contingency. Two example traces corresponding to two data points on top are shown in the bottom of panel ***C***. The red rectangle encloses the initial depolarization phase showing a faster depolarization for the lateral data point (black *) compared with the medial one (gray *). ***D***, Mean and standard deviation of the ChR2-LFP slope data from ***C***, plotted along the tonotopic axis. Slope of the linear fit to these data points is the plasticity gradient (=0.33) for this animal. Additional control data are available in Extended Data Figure 3-1.

We used the ChR2-LFP in acute slices of auditory striatum to measure the strength of the corticostriatal synapses at specific locations along the striatal tonotopic gradient (Xiong et al., 2015). To ensure that the ChR2-LFP selectively reflected the strength of cortical, rather than thalamic or other inputs to the striatum, we expressed AAV9-CAG-ChR2 in the primary auditory cortex (Fig. 3*A*). Animals were approximately five weeks old at the time of injection. After 3–5 d of recovery, the animals were trained on the tonecloud task for approximately two to three weeks until they reached the performance criterion. The duration of the training also allowed for the expression of ChR2 in the infected neurons. Once an animal reached the behavioral performance criterion (>80% for four to six consecutive sessions), we obtained acute coronal slices (Fig. 3*A*, top right) from its brain, and recorded the ChR2-LFP from the auditory striatum (Fig. 3*B*).

ChR2-LFPs evoked in these slices showed a stereotyped waveform, reminiscent of that seen in classic extracellular LFPs evoked by electrical stimulation of the Schaeffer collateral input to the CA1 region of the hippocampus (Xiong et al., 2015). Because the striatum, like the CA1 region of the hippocampus, lacks recurrent excitatory connections (Kreitzer and Malenka, 2008; Van Strien et al., 2009), this LFP can be used as a measure of synaptic efficacy (Xiong et al., 2015). As expected, the ChR2-LFP responses were evoked only in regions containing ChR2-expressing fibers (Extended Data Fig. 3-1*A*), and the magnitude of the ChR2-LFP increased with duration and strength of optical stimulation (Extended Data Fig. 3-1*B*,*C*). Pharmacological dissection of the stereotyped ChR2-LFP waveform uncovered three distinct components. The first was a very short latency light artifact (arising from the photoelectric effect); the second was the fiber volley; and the third was the synaptic response, sensitive to blockage by the AMPA receptor antagonist CNQX (Extended Data Fig. 3-1*D*). The slope of this third CNQX-sensitive component, the ChR2-LFP slope, represented a measure of corticostriatal input (Fig. 3*B*).

10.1523/ENEURO.0209-20.2021.f3-1 Extended Data Figure 3-1 Controls of ChR2-LFP recordings and measurement of ChR2-LFP slopes. ***A***, Neuronal responses to optical stimulation is absent in brain region not expressing ChR2 (somatosensory cortex). ***B***, Magnitude of ChR2-LFP increases with increase in laser power, keeping the duration of stimulation at 0.5 ms. ***C***, The magnitude of ChR2-LFP increases if duration of stimulation is increased at the highest laser power of 0.980 mW. ***D***, 30 min of slice incubation with 50 μμ CNQX abolishes the postsynaptic response of striatal neurons without affecting the depolarization of cortical fiber terminals in striatum (red) in comparison to predrug control (black). ***E***, Example of normalized ChR2-LFP slope distribution in the left auditory striatum of an animal trained on the low-right contingency. ***F***, Mean and SD of the normalized ChR2-slope data from ***E*** plotted along the tonotopic axis. Download Figure 3-1, TIF file.

**Figure 4.**
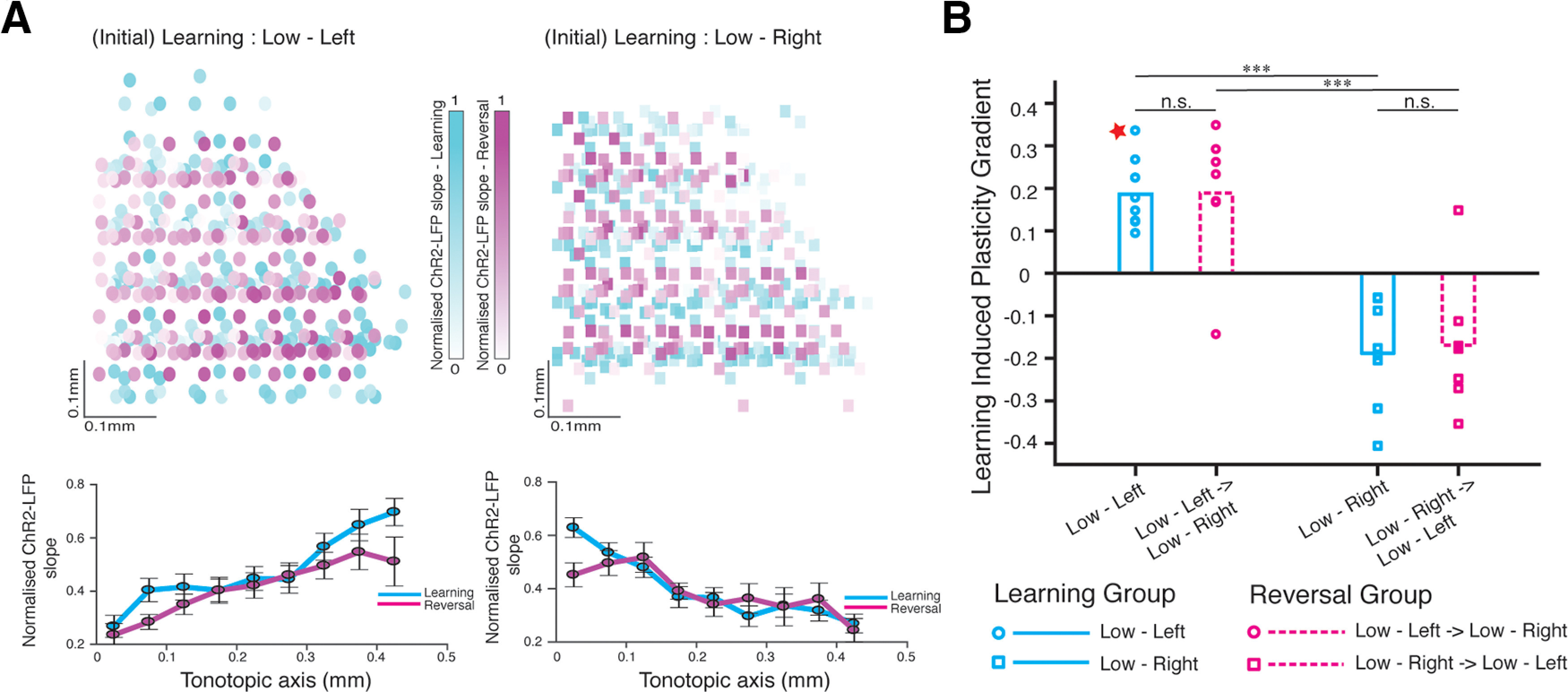
Learning-induced plasticity gradient in auditory corticostriatal circuit reflects initial learning contingency. ***A***, Top - ChR2-LFP slope after either learning alone (cyan, *n* = 8 animals) or reversal (magenta, *n* = 7 animals) from Low-Left (left) or Low-Right (right) contingency on the tonecloud task. The intensity of each point represents magnitude of normalized ChR2-LFP slope value recorded at that position of the striatal slice. Bottom - mean • SEM vs. tonotopy. Correlation coefficient of data along the tonotopic axis - Low-Left: 0.95, *p* < 2 × 10^−3^, Low-Left → Low-Right: 0.93, *p* < 1 × 10^−3^, Low-Right: −0.88, *p* < 1 × 10^−3^, Low-Right → Low-Left: ⊟0.77, *p* < 1 × 10^−2^. ***B***, Summary of learning-induced plasticity gradient from 4 groups of animals: {Learning (cyan) or Reversal (magenta)} × {Low-Left (circle) or Low-Right(square)}. Points represent individual animals. Significant differences were only observed between groups trained on opposite contingencies : Low-Left vs. Low-Right (*p* = 8 × 10^−5^) and Low-Left → Low-Right vs. Low-Right → Low-Left (*p* = 2 × 10^−3^), *** indicates *p* < 0.005; Wilcoxon Rank Sum test. The same test showed no significance between groups trained on same contingencies (n.s. = not significant; *p* > 0.05, Wilcoxon Rank Sum test). The plasticity gradient from the example animal shown in Figure 3*D* (value = 0.3) is marked here with a red star. Additional data is available in Extended Data Figure 4-1.

We recorded the ChR2-LFP from multiple positions within the left auditory striatum in each slice, and calculated the ChR2-LFP slope at each position (Fig. 3*C*, top). The slope of these ChR2-LFP values along the tonotopic axis represents the plasticity gradient for each animal (Fig. 3*D*). Based on the anatomic projections of the tonotopic inputs from the cortex to the striatum, we expected a larger ChR2-LFP in the lateral auditory striatum for animals trained on the low-left contingency, and lower for animals trained on the opposite (low-right) contingency. Figure 3*D* shows the gradient along the medio-lateral (tonotopic) axis of a single animal trained to associate low-frequency sounds with left decisions (low-left). As expected, the ChR2-LFP slope is positive (*p* = 0.0004, for positive correlation along mediolateral axis). This result was reliable: all animals (8/8) trained on the low-left contingency showed a positive slope [Fig. 4*B*, cyan bar; mean plasticity gradient = 0.19 ± 0.03 (SEM), each circle represents individual animals]. By contrast, all (8/8) animals trained on the opposite (low-right) contingency showed a negative slope [Fig. 4*B*, cyan bar; mean plasticity gradient = −0.19 ± 0.04 (SEM), each square represents individual animals]. By contrast, there was no gradient along the dorsoventral axis of the striatum, consistent with the fact that inputs along this axis are not organized tonotopically (Extended Data Fig. 4-1). Thus, training mice to associate a low-frequency or high-frequency sound with a right or left choice reliably establishes a robust gradient of synaptic strength along the tonotopic axis that faithfully represents the learned sound-action contingency (*p* = 8 × 10^−5^ between plasticity gradients of animals trained on low-left vs low-right, Wilcoxon rank-sum test, *n* = 8 in each group).

10.1523/ENEURO.0209-20.2021.f4-1 Extended Data Figure 4-1 ***A***, Normalized ChR2-LFP slope of individual animals along the tonotopic axis. The raw data as shown in Figure 4*A* have been binned in 50-μm bins. The thin lines designate individual animals trained on each task contingency and the bold lines show the mean normalized ChR2-LFP values (cyan, learning; magenta, reversal). ***B***, Summary of normalized plasticity gradient calculated along the dorsoventral axis (non-tonotopic axis) does not reflect a consistent difference between training contingencies (low-left vs low-right) or across training phases (learning vs reversal). Kruskal–Wallis test, *p* = 0.43. Download Figure 4-1, TIF file.

### The plasticity gradient established during initial training persists even after animals are trained to reverse the learned sensory-motor association

The comparable training times required by the animals to reach criterion performance during learning and reversal (Fig. 1*B*) would suggest significant changes in synaptic strengths in the neural circuit. In our simple working model, the strengthening of specific synapses from the auditory cortex to the auditory striatum is one of the circuit changes underlying the acquisition of the tonecloud task. In particular, the fact that these synapses are strengthened along the striatal tonotopic gradient, and that the sign of the gradient depends on the specific contingencies (low-left or low-right) acquired during learning (Fig. 4), suggested a simple prediction: reversing the contingencies should reverse the sign of the gradient of corticostriatal synaptic strength along the tonotopic axis.

We therefore tested the effect of reversal on corticostriatal plasticity. For each animal injected with ChR2 and trained to criterion on one contingency, we reversed the contingencies and retrained to criterion. To minimize bias, we performed recordings (blind to the training contingency).

Surprisingly, contrary to the predictions of the simple model, we found that reversing the association did not alter the sign of the plasticity gradient. The sign of the gradient was negative in animals initially trained on the low-right contingency, and remained negative even if they were subsequently trained to low-left (Fig. 4*A*, right, magenta). Similarly, sign of the gradient was positive in animals initially trained on the low-left contingency, even if they were subsequently trained to low-right (Fig. 4*A*, left, magenta). These results were robust and consistent across animals. In 100% (16/16) of animals trained without reversal, and 86% (12/14) animals trained on reversal, recordings from a single brain slice could be used to infer the animal’s initial training history. Thus, our results demonstrate that the plasticity gradient established during the initial learning persists even after subsequent learning of the opposite association.

## Discussion

We have investigated how the auditory corticostriatal circuit, critical for auditory discrimination behavior, adapts to learning and reversal of stimulus-action association. Our main findings are that (1) learning in this task establishes a strong plasticity gradient in these synapses, in a pattern that reflects the training contingencies (Fig. 3*E*; Xiong et al., 2015); and (2) training animals to reverse this association leaves this initial gradient intact. Our observations have implications for the role of corticostriatal plasticity in mediating stimulus-action associations, and more broadly, for understanding how animals adapt to an ever-changing world.

The auditory striatum, located at the caudal tip of the striatum in the rodent, receives convergent input from auditory cortex, auditory thalamus and midbrain dopamine neurons. In animals performing an auditory task, inactivation of either auditory cortical or thalamic inputs to the auditory striatum (Chen et al., 2019), or of the auditory striatum itself (Guo et al., 2018) markedly impairs performance. Optogenetic activation of cortical inputs to the striatum elicits a choice bias that depends on the frequency tuning of the stimulated site (Znamenskiy and Zador, 2013). Acquisition of the tonecloud discrimination task strengthens corticostriatal inputs during learning, in a frequency specific manner (Xiong et al., 2015). Taken together, these results suggested a simple model in which the auditory striatum couples sensory inputs to rewarded actions, mediated by the specific pattern of synaptic strength of cortical inputs to the striatum (Xiong et al., 2015; Fig. 4*B*, cyan bars).

The present results argue that this simple model is incomplete. If the auditory striatum were simply transforming auditory sensory information from the cortex into an action, then reversing the stimulus-action association would be predicted to reverse the gradient of synaptic strength in the striatum. However, we did not observe such a reversal in the plasticity gradient. Instead, we found that the gradient in synaptic strength depended only on the initial contingencies of the task the animal was trained to perform. One possible mechanism includes subtle changes in plasticity onto the different striatal cell types (e.g., direct vs indirect pathway neurons) which may change the output of the striatum. Another possibility is that other inputs to the auditory striatum are involved during the behavioral reversal. In principle, the thalamic inputs to auditory striatum might play a role in transforming auditory sensory information into action, although the fact that the major thalamic inputs to the striatum arise from the dorsal medial geniculate nucleus (Chen et al., 2019), an area in which neurons are not well-tuned to sound, argues against these inputs providing the necessary frequency-specific information. Furthermore, electrophysiological recordings in behaving mice show that the activity of auditory striatal neurons was only modestly influenced by the animals’ choices (Guo et al., 2018). Taken together, these observations reveal the limitations of our previous model, and suggest that the locus for transforming sensory inputs to actions might lie outside of the auditory striatum.

Our results indicate that rather than overwriting the initial memory trace, animals may keep this initial trace intact. This strategy may prevent what in artificial neural network research has been termed “catastrophic forgetting” (McCloskey and Cohen, 1989): the loss of old memories on acquisition of new ones. However, in some cases, such as the alignment of auditory and visual maps, it appears that several distinct alignments can co-exist within a single circuit (Knudsen and Brainard, 1995; Feldman and Knudsen, 1997; Linkenhoker et al., 2005). Whether or not a similar preservation of overlapping stimulus-action associations is possible in the auditory or other sensory striatum remains unexplored. Chronic electrophysiological recordings from striatal neuronal populations during flexible behaviors might be used to determine whether striatal cells can respond differently to the same stimulus under different task/behavioral contexts. However, a more general solution to the catastrophic forgetting problem may be to recruit new brain circuits when a new memory is added. In the case of the reversal of stimulus-reward associations (Schoenbaum et al., 2009), several brain areas have been implicated, including the orbitofrontal cortex, the medial prefrontal cortex, the basolateral amygdala and the ventral striatum (Schoenbaum et al., 1999; Schoenbaum and Setlow, 2003; Johnson et al., 2016). Understanding how these different brain circuits coordinate with regions such as the auditory striatum that show such strong representations of stimulus action associations represents an important challenge for future work. Unraveling the circuit and synaptic basis of reversal learning in this task may provide a foundation for understanding how both natural and artificial systems adapt to new situations while avoiding catastrophic forgetting.

## Synthesis

Reviewing Editor: Christine Portfors, Washington State University Decisions are customarily a result of the Reviewing Editor and the peer reviewers coming together and discussing their recommendations until a consensus is reached. When revisions are invited, a fact-based synthesis statement explaining their decision and outlining what is needed to prepare a revision will be listed below. The following reviewer(s) agreed to reveal their identity: Luan Castro Tonelli.

The reviewers agree that the manuscript provides substantial advances on the understanding of corticostriatal plasticity in learning processes. The findings help to understand neural basis of learning in adaptive behaviors. While the paper is well written and provides important information, the over-interpretation of the negative results reduces enthusiasm for the paper. The authors agree that the main criticisms of the manuscript can be addressed by revising the manuscript and not providing any additional experimental evidence.

Reviewer 1 comments:

In this study, the authors explored the effect of behavioral reversal on auditory corticostriatal plasticity established after initial learning. The authors first show that mice can learn a two-alternative force choice, auditory-motor reversal task. They then replicated previous results showed in rats by Xiong et al. (2015): 1) auditory corticostriatal projections are tonotopically organized and 2) there is a learning-induced gradient of corticostriatal synaptic strength along the tonotopic gradient in the striatum. These results while confirmatory of previous studies in rats are an important contribution to the literature demonstrating general principles across two rodent species. Finally, in new work, they showed that reversal learning does not appear to impact the ChR2-stimulated LFP slope plasticity gradients along the tonotopic axis in the striatum. Overall, this study poses an interesting question: is synaptic plasticity in the tonotopic corticostriatal gradient impacted by task contingency reversal as distinct from initial learning? The main result is somewhat surprising as well - the lack of change in ChR2-LFP slope after contingency reversal. The authors developed a nice reversal behavioral paradigm in mice and used a clever physiological method to measure synaptic efficacy that leverages optogenetics and simple LFP recordings similar to Xiong et al., 2015. While generally a nice paper, there are some concerns that moderately dampen enthusiasm, including an over-interpretation of their negative result:

1) Figure 4 represents the main result of the study and could contain more information

a. Statistics are needed for the changes in ChR2-LFP slopes along the tonotopic axes (Fig. 4A). Seems as though there is a reduction in the slope at both extremes of the tonotopic axis for ‘low-left’ and near 0 for ‘low-Right’. This could be relevant insofar as the trained stimuli likely reflect the extremes of the tonotopic gradient.

b. It would be helpful to see the learning-induced plasticity gradient (Fig. 4A, bottom) for each animal and/or slice; this would either confirm the mean result or, if there were two clear distributions, might suggest something more complicated is going on.

c. In Fig. 4B, it would be interesting to have a sense of what a slope of e.g. 0.1 means: what would be a distribution of slopes from naïve mice? (Data from Xiong et al., 2015, Extended Data Figure 6).

d. Rather than just comparing the slopes of the linear fit to this data (Figure 4B), it would be valuable to compare the normalized ChR2-LFP slopes obtained after initial learning and reversal at the location of the respective frequency range of the two stimuli. This would more directly link plasticity changes (or their absence) to stimulus identity.

e. The lack of a significant difference between the learning and reversal groups is the primary conclusion of the paper. Importantly, the absence of evidence does not indicate an evidence of absence. Their optogenetic-LFP assessment of synaptic plasticity is powerful but limited; using LFP recordings provides bulk activity patterns upon ChR2 stimulation. How might an initial memory trace and one with reversed contingencies co-exist in the same circuit? One possibility would be cell-specific activation patterns that would be obscured by bulk measurements. Overall, while the initial hypothesis was that the plasticity gradient would be reversed after reversal, it need not be so simple. Overall, the strong claim that the ‘locus for transforming sensory inputs to actions is likely outside of the auditory striatum’ cannot be made with only negative results. I would strongly suggest a richer description of the alternatives; of which one is that the transformation occurs outside of the auditory striatum.

f. Overall, I think their data reject the simple framework provided in Xiong et al., 2015; but only as it relates to using the ‘specific pattern of synaptic strength of cortical inputs to the striatum’ as the principle measure of learning and plasticity in the auditory striatum. They cannot reject, as yet, the notion that the auditory striatum couples sensory inputs to rewarded actions because that may be reflected in the circuit in other ways.

g. The title of the paper, for example, seems like the most appropriate implication of their results. Even if there are other elements that change within the auditory striatum (that they do not measure), it remains valuable to know that the plasticity gradient established by initial learning does persist after reversal.

2) Figure 2 is surprisingly devoid of statistics, number of slices/animals (for 2C-E) and the images from B are very grainy and no quantification or statistics are provided.

3) Figure 3 should include summary data since they are establishing that they can do in mice what has already been done in rats. They indicate an n=8; is that mice or slices? Also, need to justify the use of non-parametric tests.

Minor comments:

- Auditory stimuli used in the task are not described in the method section "Behavioral training and apparatus" (frequency range, sound statistics, duration).

- Lines 309-310: ‘Because the striatum, like the hippocampus, lacks recurrent excitatory connections’ as stated is incorrect. The CA3 region of the hippocampus has very high density of recurrent collaterals (the auto-associative network). CA1 recurrent loops, however, are limited and restricted to one septotemporal pole level (van Strien et al., 2009). This should at least be adjusted to say ‘like the CA1 region of the hippocampus’.

- The authors did not mention normality testing. Usage of non-parametric tests should be justified (lines 215-220).

- Figure 4A top: I would advise the authors to increase the contrast of the (green and pink) color scales because the changes are very hard to notice.

- Authors did not mention the post-hoc test used (Extended Figure S3, kruskal wallis test).

Reviewer 2 comments:

The manuscript is well written, having excellent references and methods that substantiate its findings.

I would like to suggest a couples of changes or adjustments:

Abstract:

Line 16: add a sentence explaining the implications of your findings for further investigations on the topic.

Introduction:

Lines 72 to 75: ..."a different sub-part of substantia nigra compared to the anterior striatal"... this part is confusing, and it needs to be adjusted accordingly.

Materials and Methods

Line 131 and 132: provide a reference for the frequencies chosen and explain briefly why?

Line 138: "water-deprived" , please explain how deprived the animals were for future replications of the study and animal care.

Results:

Images depicts quite nicely the study-design and it helps the reader to understand the findings.

Discussion:

I appreciate the way the discussion was written, however, I would like to see a more extended and elaborated discussion explaining your findings thoroughly and further steps to continue this research.
