# Supplementary figures and images for: Corticostriatal Plasticity Established by Initial Learning Persists after Behavioral Reversal
Source: eNeuro. 2021 Mar 10;8(2):ENEURO.0209-20.2021. doi: 10.1523/ENEURO.0209-20.2021 (PMC7986528; doi:10.1523/ENEURO.0209-20.2021)

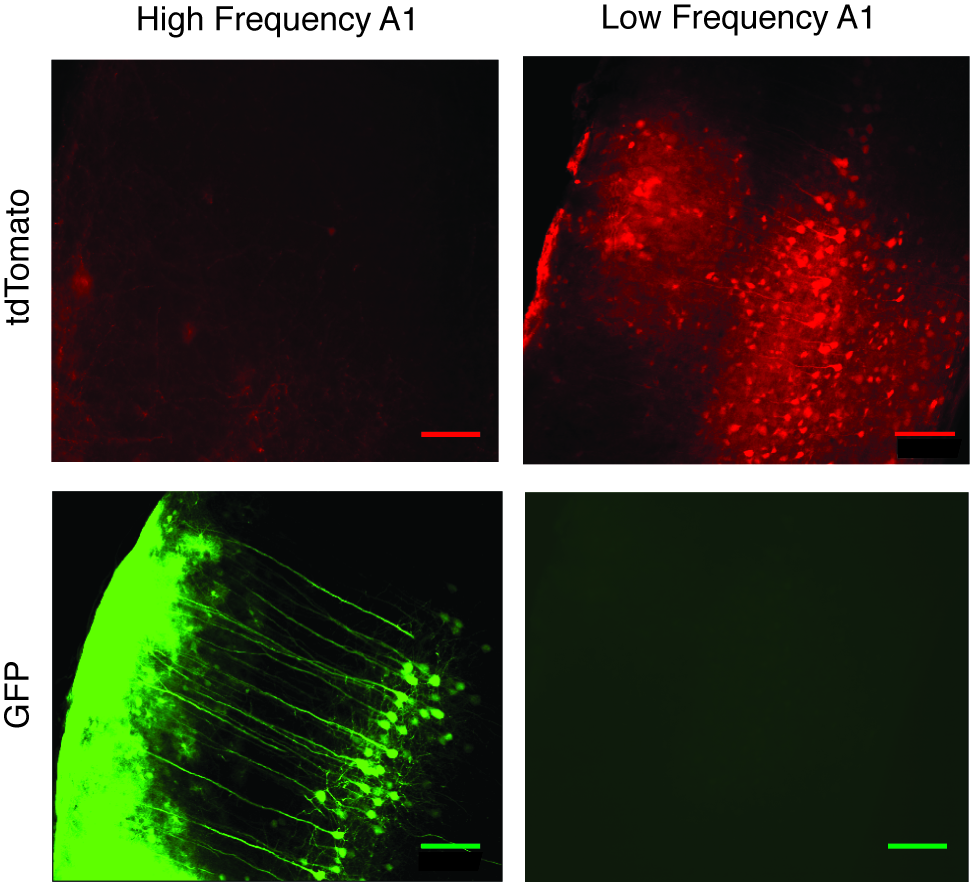

Supplement: Extended Data Figure 2-1 — Injection site in primary auditory cortex confirming expression of tdTomato (top) and GFP (bottom). These images confirm little to no overlap of viral infections at the cortical injection site. Scale bar: 500 μm. Download Figure 2-1, TIF file. [file enu-eN-NWR-0209-20-s01.tif]

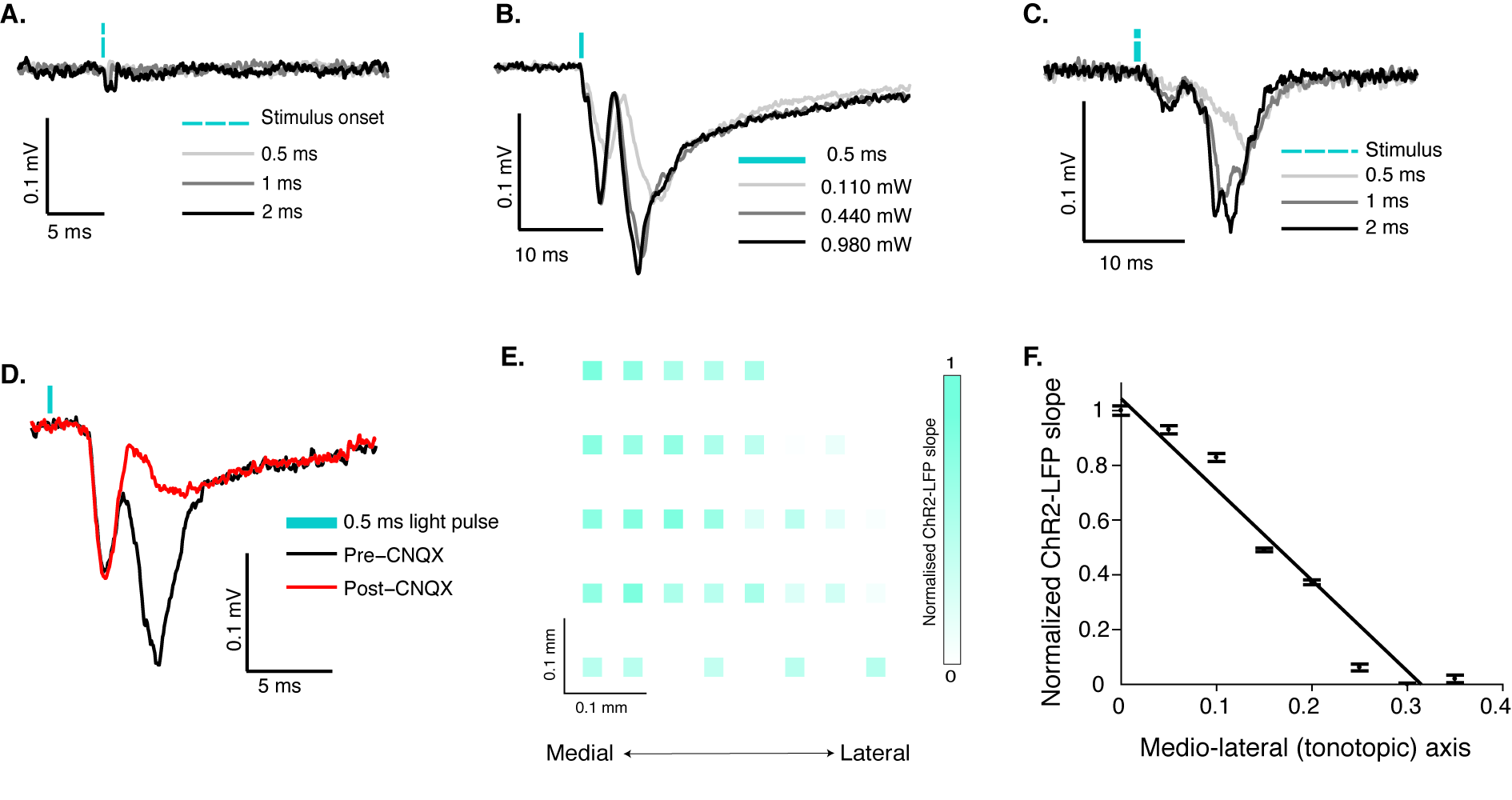

Supplement: Extended Data Figure 3-1 — Controls of ChR2-LFP recordings and measurement of ChR2-LFP slopes. A, Neuronal responses to optical stimulation is absent in brain region not expressing ChR2 (somatosensory cortex). B, Magnitude of ChR2-LFP increases with increase in laser power, keeping the duration of stimulation at 0.5 ms. C, The magnitude of ChR2-LFP increases if duration of stimulation is increased at the highest laser power of 0.980 mW. D, 30 min of slice incubation with 50 μμ CNQX abolishes the postsynaptic response of striatal neurons without affecting the depolarization of cortical fiber terminals in striatum (red) in comparison to predrug control (black). E, Example of normalized ChR2-LFP slope distribution in the left auditory striatum of an animal trained on the low-right contingency. F, Mean and SD of the normalized ChR2-slope data from E plotted along the tonotopic axis. Download Figure 3-1, TIF file. [file enu-eN-NWR-0209-20-s02.tif]

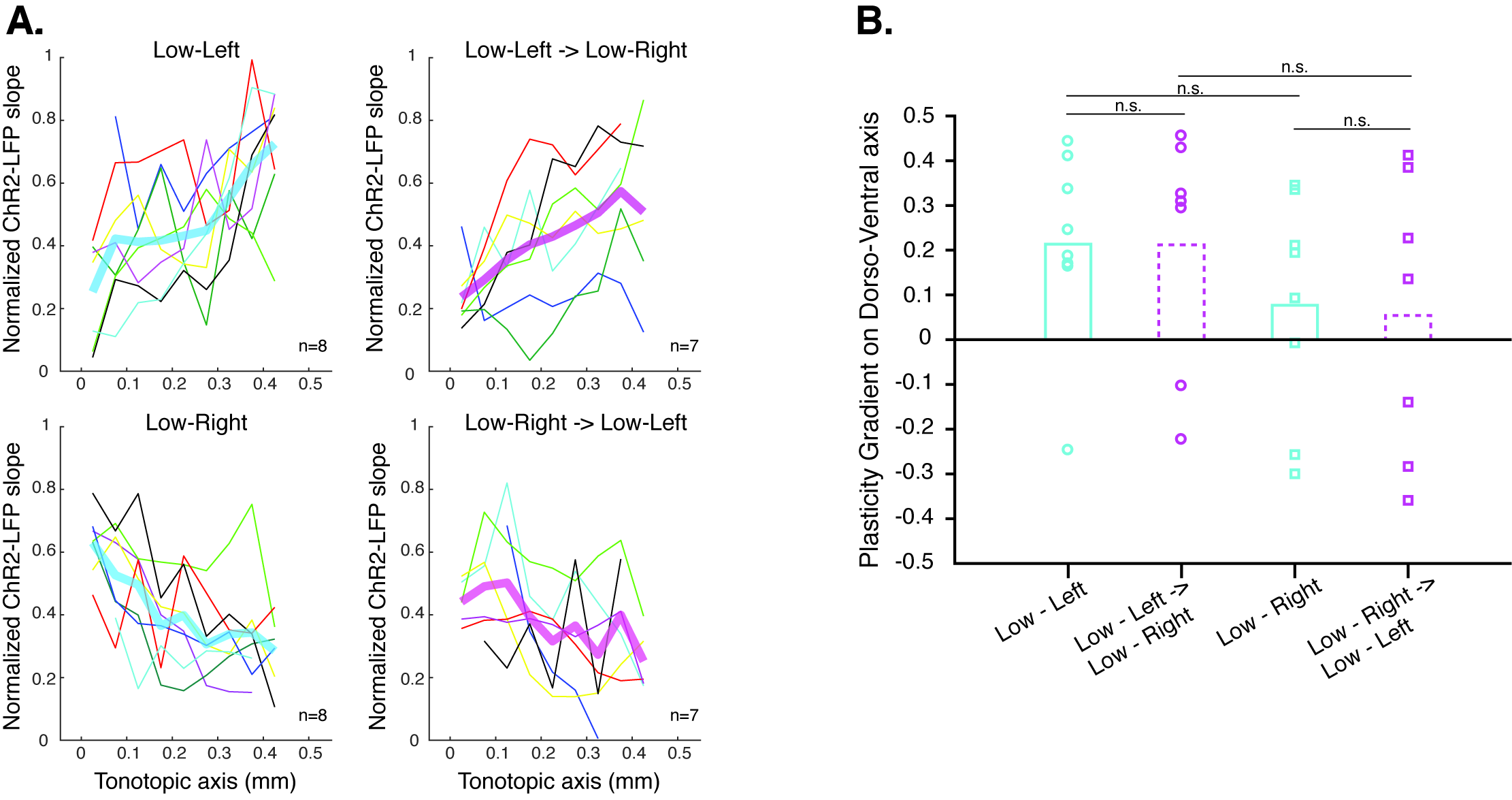

Supplement: Extended Data Figure 4-1 — A, Normalized ChR2-LFP slope of individual animals along the tonotopic axis. The raw data as shown in Figure 4A have been binned in 50-μm bins. The thin lines designate individual animals trained on each task contingency and the bold lines show the mean normalized ChR2-LFP values (cyan, learning; magenta, reversal). B, Summary of normalized plasticity gradient calculated along the dorsoventral axis (non-tonotopic axis) does not reflect a consistent difference between training contingencies (low-left vs low-right) or across training phases (learning vs reversal). Kruskal–Wallis test, p = 0.43. Download Figure 4-1, TIF file. [file enu-eN-NWR-0209-20-s03.tif]
